# Hazards Constitute Key Quantities for Analyzing, Interpreting and Understanding Time‐to‐Event Data

**DOI:** 10.1002/bimj.70057

**Published:** 2025-06-06

**Authors:** Jan Beyersmann, Claudia Schmoor, Martin Schumacher

**Affiliations:** ^1^ Institute of Statistics, Ulm University Ulm Germany; ^2^ Clinical Trials Unit, Faculty of Medicine and Medical Center University of Freiburg Freiburg Germany; ^3^ Institute of Medical Biometry and Statistics Faculty of Medicine and Medical Center University of Freiburg Freiburg Germany

**Keywords:** collider effect, hazard ratio, independent censoring, intention‐to‐treat, time‐varying effect

## Abstract

Censoring makes time‐to‐event data special and requires customized statistical techniques. Survival and event history analysis therefore builds on hazards as the identifiable quantities in the presence of rather general censoring schemes. The reason is that hazards are conditional quantities, given previous survival, which enables estimation based on the current risk set—those still alive and under observation. But it is precisely their conditional nature that has made hazards subject of critique from a causal perspective: A beneficial treatment will help patients survive longer than had they remained untreated. Hence, in a randomized trial, randomization is broken in later risk sets, which, however, are the basis for statistical inference. We survey this dilemma—after all, mapping analyses of hazards onto probabilities in randomized trials is viewed as still having a causal interpretation—and argue that a causal interpretation is possible taking a functional point of view. We illustrate matters with examples from benefit–risk assessment: Prolonged survival may lead to more adverse events, but this need not imply a worse safety profile of the novel treatment. These examples illustrate that the situation at hand is conveniently parameterized using hazards, that the need to use survival techniques is not always fully appreciated and that censoring not necessarily leads to the question of “what, if no censoring?” The discussion should concentrate on how to correctly interpret causal hazard contrasts and analyses of hazards should routinely be translated onto probabilities.

## 1 Introduction

Hazards are the backbone of the analysis of time‐to‐event data. Cox's proportional hazards model and the Kaplan–Meier estimator, a transformation of Nelson–Aalen's cumulative hazard estimator, are ubiquitous. The reason is censoring. Times‐to‐event are typically incompletely observed and hazards are the identifiable quantities under rather general censoring schemes. Hazards are identifiable, because they are conditional quantities given previous survival. As a consequence, cumulative hazard increments can be estimated based on the individuals currently at risk, that is, alive and under observation.

For precisely the same reason, being conditional quantities given previous survival, the interpretation of hazards has more recently been questioned from a causal perspective, in particular following the influential paper by Hernán (2010), and the critique is even put forward in the context of randomized interventions: Conditioning on previous survival is conditioning on a postrandomization event and therefore breaks randomization. This appears to be a vicious circle, at least for randomized trials with a time‐to‐event outcome: The aim is to detect the causal effect of the randomized intervention, the data will arguably be censored, hence hazard‐based statistical techniques are required, but such techniques condition on a postrandomization event.

To complicate matters further, much of the literature discussing this issue may raise the impression that the debate is about hazard ratios as a measure of effect (e.g., Aalen et al. 2015; De Neve and Gerds 2020; Hernán 2010; Martinussen 2022), but, as, for example, Martinussen (2022) clearly states, the core of the argument is the hazard function itself. The point is subtle: There is no debate that contrasts of unconditional survival probabilities following a randomized intervention have a causal interpretation. However, there is a deterministic one‐to‐one mapping between hazard function and survival function, and the question arises why a causal contrast of the latter should not translate into a causal contrast of the former, and vice versa. In fact, Hernán (2010) suggests, first, a statistical analysis of hazards and, second, to transform the first analysis onto the unconditional probability scale. This suggests that a causal contrast should be somehow “hidden” in the hazards analysis. A related statement is by Martinussen (2022), who claims that the hazard ratio “cannot be given a causal interpretation when interpreted as an HR,” that is, as a hazard ratio. In other words, contrasts of hazards may lend themselves to a causal interpretation, but such an interpretation on the hazard scale is in doubt.

One worry is that such subtleties may be lost in translation (e.g., Murray et al. 2021; Li et al. 2022) resulting in rather sweeping statements that hazard ratios do not lend themselves to a causal interpretation. A more fundamental concern is related to how we define a causal effect in statistics: Any hope for a general philosophical theory of causality has largely been destroyed since David Hume's *A Treatise of Human Nature* (Hume 1896), see Russell (2004) for a very readable account and Otsuka (2023) for a philosophical view on statistics including questions of induction and causality. Randomized controlled trials circumvent such theoretical problems by randomization, and causal inference translates this approach to observational data by trying to detect effects had exposure been randomized. Throughout, this will be our approach to formalizing a causal efect: Any difference or contrast between the probability distributions of groups randomized to different interventions displays a causal intervention effect. In a time‐to‐event context, probability distributions are typically parameterized by a possibly multivariate hazard measure, and a causal effect may contrast the interventional distributions as such or aspects thereof, for example, medians or, as we will argue, hazard functions. That is, following the point of view that any difference between probability distributions following randomization has a causal interventional interpretation, the question arises whether we can intervene on a population now, in a randomized fashion, to impact a future hazard function of that population. For example, can we randomize a population to an intervention where the aim is to leave the population's hazard function for an adverse event unchanged, but to replace its hazard function for the primary disease outcome by a smaller hazard function? If so, the question is why not express matters in terms of hazard contrasts? However, the debate around hazard ratios as a contrast suggests a negative answer—which is puzzling, because we have respected the temporal sequence of first cause, then effect.

The aim of this paper is to argue that hazard contrasts, including but not limited to hazard ratios, time‐constant or time‐varying, can be given a causal interpretation if interpreted in a functional sense. That is, we argue that hazard contrasts are to be interpreted as comparing hazard functions rather than hazards at a fixed point in time. We demonstrate that the issue of conditioning on a postrandomization event, resulting in so‐called “collider bias,” vanishes from a functional point of view. We argue that this does not dispel the subtleties present when working with hazards, and in line with much of the current literature in the field, we strongly support that hazard analyses are much more routinely translated onto the probability scale, see Andersen (2022) for a recent review. We demonstrate the need for the latter by two examples from benefit–risk assessment; there, we also argue that, first, the need to account for censoring of time‐to‐event data is sometimes less well appreciated than is desirable—a worry in its own right, but perhaps even more so against the background of the “causal hazard ratio” debate. And second, we argue that, say, a formalization of an effective drug that is safe in comparison may naturally be achieved using hazards, where both efficacy and safety are evaluated in comparison with a control group.

One cornerstone of the discussion has been the question of how to interpret time‐varying hazard ratios, traditionally viewed as one method to investigate time‐varying effects. Following Hernán (2010), a critique of, for example, period‐specific hazard ratios has been that, first, hazards are conditional quantities that break randomization and, second, even constant hazard ratios within different time periods must consequently not be interpreted as time‐varying effects. However, “collider bias” affects hazards whatever the contrast between groups, while period‐specific reporting produces its own selection. These are two distinct issues. If hazards can be used to understand the evolution of time‐to‐event data, their analysis should shed some light on time‐varying effects and we offer a suggestion.

The remainder of the paper is organized as follows: Section 2 reiterates why hazards are the identifiable quantities in the presence of censoring. Here, we also explain a counting process–type of requirement on censoring needed to ensure identifiability that is much more general than the common random censorship assumption (possibly given covariates). We also give a two‐line proof why so‐called competing events may be coded as censoring for a hazard analysis, which in turn demonstrates that censoring is not restricted to inference for causal “what, if no censoring?” scenarios. Another example of such more general “independent censoring” are event‐driven trials, common in clinical research. Both of these more general censoring schemes are relevant for our examples. Of the following three sections, Section 3 argues why there can be a causal interventional effect on hazard functions, Section 4 investigates how a causal contrast plays out in potentially time‐varying hazard ratios and Section 5 considers a situation where the hazard measure is multivariate. To this end, Section 3 explains “collider bias” as a consequence of conditioning on previous survival, which is at the core of the debate, and how “collider bias” vanishes from a functional point of view. In this section, we also argue that “collider bias” is really a “collider effect” and that this form of “collider effect” is not tied to (or may even have little to do with) time‐to‐event data. Section 4 more specifically considers hazard ratios, either constant or piecewise constant. Constant hazard ratios may be given an interpretation as a causal contrast on the probability scale (e.g., De Neve and Gerds 2020), while time‐varying hazard ratios have traditionally been interpreted as a time‐varying effect. The latter interpretation has been challenged in the current debate (e.g., Hernán 2010; Martinussen et al. 2020). We will argue that, for example, piecewise constant hazard ratios do carry information on how treatment works, but that a “time‐varying effect” may be a more elusive concept than suspected at first glance. Section 5 considers two examples from benefit–risk assessment mentioned earlier and a discussion is offered in Section 6. We neither report simulation studies nor guidance for software support. For the latter, any software analyzing hazards, including but not limited to fitting proportional hazards models, applies. For the former, a simulation study would, for example, investigate a method to fit a proportional hazards model and report, say, empirical bias and variation of the method under investigation. The focus of this work, however, is the interpretation of such an analysis.

## 2 Why We Need Hazards

This section is organized as follows: We begin by arguing that censored time‐to‐event data require specialized techniques. This is, of course, the reason why survival analysis is a field in its own right, but Section 5 demonstrates that the need to employ such specialized techniques is not necessarily acknowledged in practice. We then find that hazards remain identifiable assuming random censoring, that is, stochastic independence of time‐to‐event and censoring time. Arguably, a large part of the literature assumes random censoring, possibly given covariates, but we recapitulate that a weaker assumption, called independent censoring here, suffices to guarantee identifiability. One example from randomized trials is event‐driven censoring, where time‐to‐event and censoring time are stochastically dependent and censoring even informative in that it is driven by the distributions of time‐to‐event. This type of censoring occurred in the examples of Section 5. We finally give a simple proof of why a competing event acts as independent censoring, again of relevance for Section 5, but, as we also explain, this example differs from the previous examples of censoring in that it does not lend itself to a “what, if?” interpretation. Against a more general causal background, this observation is of relevance as it helps to distinguish the overarching question at hand—can we have a causal intervention effect on a hazard function, possibly in the presence of multiple hazards?—from notorious causal misinterpretations of competing events analyses.

To begin, we consider a typical situation when analyzing survival data. We are interested in estimating the distribution of time‐to‐event, possibly within, say, arms of a randomized trial, which is complicated by the presence of censoring. To formalize matters, consider a proper survival time T∈(0,∞), where “proper” means that T is finite with probability one, for example, if T denotes time‐to‐death. Observation of T shall be subject to right‐censoring by C∈(0,∞], which includes the uncensored case where C=∞ with probability one. The observable time‐to‐event data are replicates of

T∧C,1(T≤C),

where ∧ denotes the minimum and 1(·) the indicator function. Assume the simple scenario where we have n i.i.d. replicates of the observable time‐to‐event data with the ith replicate indexed by i, i∈{1,⋯,n}. Applying standard statistical methods to this set‐up will implicitly target parameters that typically depend on the joint distribution of T and C, but we are usually interested in the distribution of T, while that of C is a nuisance. To illustrate, the proportion of observed events in [0,t],

(1) $$\frac{\sum_{i=1}^{n}1\left(T_{i}\wedge C_{i}\leq t,T_{i}\leq C_{i}\right)}{n}$$

does not estimate the probability of an event in [0,t] but rather the smaller probability of an observed event in [0,t], that is, P(T≤t,T≤C). The analysis of adverse events in clinical trials with time‐to‐event efficacy outcomes is one example where reporting the proportion of observed events is common, see the recent effort of Stegherr, Beyersmann, et al. (2021), Stegherr, Schmoor, et al. (2021), and Rufibach et al. (2023) to remedy this. A possible explanation is that such bias is rarely explicitly stated in the textbook literature, a notable and clear exception being Nishiyama (2021); see also the research tutorial by Clark et al. (2003).

Assume—for the time being, but soon to be relaxed—random censoring, that is, assume that survival and censoring time are stochastically independent, T⊥⊥C, and also assume T to be absolutely continuous such that its hazard function α(t) exists,

(2) $$\alpha \left(t\right)=\lim_{\Delta t↘0}\frac{P\left(T\in [t,t+\Delta t)\;|\;T\geq t\right)}{\Delta t}$$

(3) $$\Leftrightarrow \alpha \left(t\right)\;dt=P\left(T\in [t,t+\;dt)\;|\;T\geq t\right),$$

where we have written dt for the length of an infinitesimally small time interval. The reason why we need hazards is that we may re‐express the right‐hand side of Equations (2) and (3) in terms of the observable data. We have that

(4) $$\begin{array}{cc} P\left(T\in [t,t+\;dt)\;|\;T\geq t\right) & =P\left(T\in [t,t+\;dt)\;|\;T\wedge C\geq t\right)\\ & =P\left(T\in [t,t+\;dt),T\leq C\;|\;T\wedge C\geq t\right) \end{array}$$

(5) $$\begin{array}{cc} & \;=P(T\wedge C\in [t,t+\;dt),\\ & \;T\leq C\;|\;T\wedge C\geq t), \end{array}$$

where the first line follows because T⊥⊥C and the second line because no two events happen at precisely the same time. Equation (4) conditions on being at risk, 1(T∧C≥t)=1, and considers the probability of an observed event during the next infinitesimally small time step. Thus, (4) and (5) motivate to estimate the cumulative hazard $A\left(t\right)=\int_{0}^{t}\alpha \left(u\right)\;du$ using the Nelson–Aalen estimator,

$$\hat{A}\left(t\right)=\sum_{u\leq t}\frac{\sum_{i=1}^{n}1\left(T_{i}\wedge C_{i}=u,T_{i}\leq C_{i}\right)}{\sum_{i=1}^{n}1\left(T_{i}\wedge C_{i}\geq u\right)},$$

where the sum is over all unique, observed event times u in [0,t]. The Kaplan–Meier estimator of the survival function P(T>t) then simply is

$$\begin{array}{cc} \hat{P}\left(T>t\right) & =\prod_{u\leq t}\left(1-\frac{\sum_{i=1}^{n}1\left(T_{i}\wedge C_{i}=u,T_{i}\leq C_{i}\right)}{\sum_{i=1}^{n}1\left(T_{i}\wedge C_{i}\geq u\right)}\right)\\ & =\prod_{u\leq t}\left(1-\Delta \hat{A}\left(u\right)\right), \end{array}$$

that is, the product over one minus the additive increments of $\hat{A}\left(t\right)$, the latter viewed as function of time, that is, a stochastic process. This illustrates, first, that the hazards are the identifiable quantities in the presence of censoring and, second, that we may recover probabilities by transforming hazard functions. We return to the latter relationship in Section 3 and focus on identifiability in this section.

Now, using hazards, the counting process approach to time‐to‐event data neither restricts itself to simple random censorship, T⊥⊥C, nor to time‐to‐death (or some other proper survival time) as the outcome. We write N(t)=1(T∧C≤t,T≤C) for the individual counting process of an observed death event in [0,t] and $F_{t-}$ for the past generated by the observable data on [0,t),

1(T∧C≤s)and1(T∧C≤s,T≤C)fors∈[0,t),

that is, given the past $F_{t-}$ up to, but excluding time t, we know about a unit's observed death or censoring events before time t, if any. Consequently, given $F_{t-}$, we know whether a unit is at risk, 1(T∧C≥t), just prior t. We may now rewrite (4) as

(6) $$P(dN\left(t\right)=1\;|\;F_{t-})=1\left(T\wedge C\geq t\right)\cdot \alpha \left(t\right)\;dt,$$

with dN(t) the increment of the process on [t,t+dt). The idea is now to consider some counting process of some event type—possibly recurrent, possibly subject to competing events—such that the conditional probability of an observed event in [t,t+dt) given the past is equal to an at‐risk function times some hazard (or intensity), where the hazard or the cumulative hazard is the quantity to be estimated, while the at‐risk function accounts for observational restrictions such as censoring. We first turn to the question which kind of censoring preserves this structure to ensure identifiability of hazards, still considering a proper survival time T∈(0,∞) as laid out earlier.

In the counting process literature (Andersen et al. 1993; Aalen et al. 2008), the weaker assumption of independent censoring—as opposed to the strict assumption of random censoring, possibly given covariates—ensures such identifiability. (Note that the term independent censoring may not be ideal, as it may be interpreted as assuming stochastic independence of time‐to‐event and time‐to‐censoring.) The idea of this concept is to first identify a stochastic model that allows to define the target parameters of inference. Next one allows for virtually any censoring process that does not impact the target parameters if one additionally conditions on the censoring information. The latter is not unlike formalizing that a covariate has no impact in a regression model: Additionally including the covariate in a linear predictor should have regression coefficient zero. So, first, consider a past, say ${\tilde{F}}_{t-}$, that allows to define the parameter to be estimated. In the example above, ${\tilde{F}}_{t-}$ need only be generated by

1(T≤s)fors∈[0,t),

that is, ${\tilde{F}}_{t-}$ encodes knowledge about a unit's past death event in [0,t), if any, but censoring by C is not included. The reason for not including censoring by C is that only knowledge about the survival time T is needed to define the target parameter α(t), as we have

(7) $$P(T\in \left[t,t+\;dt\right)\;|\;{\tilde{F}}_{t-})=1\left(T\geq t\right)\cdot \alpha \left(t\right)\;dt.$$

The relation between Equations (6) and (7) is that the right‐hand side of each equation displays the same hazard α(t) but different leading indicator functions reflecting situations with (Equation 6) and without censoring (Equation 7).

Independent censoring now holds, if the additional knowledge of censoring does not change (7). That is, if, on the left‐hand side of (7), we do not only condition on ${\tilde{F}}_{t-}$, but enlarge this information by the additional knowledge of censoring, the right‐hand side of (7) must not change. A straightforward calculation for conditional expectations then shows that (6), that is, identifiability of hazards from the observable data, follows (e.g., Aalen et al. 2008, section 2.2.8).

Of course, random censoring is also independent censoring in this sense and little has been gained so far. But consider now as an example event‐driven censoring, a common censoring scheme in clinical trials, where the data are to be analyzed once a planned number of events has been observed in calendar time. To illustrate, consider a toy example with n=2 units entering the study at the same calendar time and censoring induced when m=1 event has been observed. We assume that the event times $T_{1}$ and $T_{2}$ are stochastically independent, but the observable data are $T_{1}\wedge T_{2},1\left(T_{1}\leq T_{2}\right)$ for unit 1 and $T_{1}\wedge T_{2},1\left(T_{2}\leq T_{1}\right)$ for unit 2. Clearly, neither are the censored data stochastically independent across units nor is censoring random. However, censoring is independent in the aforementioned sense: The additional censoring knowledge amounts to knowing about the other person, which does not change personal hazards! This result is well documented when units are put on trial at the same time (Aalen et al. 2008; Andersen et al. 1993), but it is a surprisingly recent result for the common situation of event‐driven trials with entry staggered over calendar times, see Rühl et al. (2023).

So far, the aim of the analyses could be interpreted as identifying hazards, had there been no censoring. But independent censoring is not restricted to such “what, if?” considerations. To illustrate, consider time‐to‐cardiovascular‐death, a common outcome in trials on patients with diabetes. Still writing T for time‐to‐death, we consider the additional mark ε∈{1,2}, with ε=1 for cardiovascular death, and ε=2 for other death. The event types ε∈{1,2} are also called “competing events.” The hazard α(t) of T now splits into two event‐specific hazards

$$\alpha_{j}\left(t\right)=\lim_{\Delta t↘0}\frac{P\left(T\in [t,t+\Delta t),\varepsilon =j\;|\;T\geq t\right)}{\Delta t},\;j\in \left\{1,2\right\}.$$

Using arguments analogous to above, one finds event‐specific Nelson–Aalen estimators

$${\hat{A}}_{j}\left(t\right)=\sum_{u\leq t}\frac{\sum_{i=1}^{n}1\left(T_{i}\wedge C_{i}=u,\varepsilon_{i}=j,T_{i}\leq C_{i}\right)}{\sum_{i=1}^{n}1\left(T_{i}\wedge C_{i}\geq u\right)},$$

which treat observed type 2 events as censored in the calculation of ${\hat{A}}_{1}\left(t\right)$ and observed type 1 events as censored in the calculation of ${\hat{A}}_{2}\left(t\right)$. This is not a mere computational trick. Consider, for example, the counting process of observed type 1 events, observation of which may be censored by an observed type 2 event. Identifiability of the cumulative event‐specific hazard of type 1 follows if censoring by an observed type 2 event is independent censoring. To show this, we need to demonstrate that the additional knowledge of such censoring does not change the conditional probability of a type 1 event. This holds true, because, tracking a unit's past, we know about the unit's survival and event status, and, hence, the knowledge of such censoring is not additional—it is already known and nothing changes. A formal proof is in Example III.2.6 of Andersen et al. (1993), but the main idea is easily explained, extending the arguments leading to (4). Introduce the time of censoring by a type 2 event,

ϑ=inf{t:T≤t,ε=2},

where, as usual, we define the infimum of the empty set as infinity. Hence, ϑ=T if ε=2, but ϑ=∞ if ε=1. The interpretation is that “other death” does not (never) happen, if a person has died from cardiovascular reasons. Note that ϑ≥T. Now,

(8) $$\begin{array}{ccc} \alpha_{1}\left(t\right)\;dt & = & P\left(T\in [t,t+\;dt),\varepsilon =1\;|\;T\geq t\right)\\ & = & P\left(T\in [t,t+\;dt),\varepsilon =1\;|\;T\wedge \vartheta \geq t\right)\\ & = & P\left(T\in [t,t+\;dt),\varepsilon =1,T\leq \vartheta \;|\;T\wedge \vartheta \geq t\right), \end{array}$$

where the first line is analogous to (3), and the second and third lines follow because ϑ≥T. A more formal argument realizes that ϑ is a stopping time, see Example III.2.6 of Andersen et al. (1993); we also note that (8) is in line with the presentation of Andersen and Ravn (2024) that the additional knowledge of censoring must not alter hazards. The main point here is the formal similarity between Equations (4) and (8), with (8) now demonstrating identifiability of the (cumulative) event‐specific hazard by treating the other competing event as censoring. However, and in contrast to (4), T¬⊥⊥ϑ and (8) does not lend itself to “what, if?” considerations as explained below.

For the record, we also note that not only does the “all‐events” Nelson–Aalen estimator split into its event‐specific parts ${\hat{A}}_{1}\left(t\right)$ and ${\hat{A}}_{2}\left(t\right)$—one minus Kaplan–Meier also splits into (so‐called) Aalen–Johansen estimators of the cumulative event probabilities of outcomes ε=1 and ε=2. We have

$$\begin{array}{ccc} 1-\hat{P}\left(T>t\right) & = & 1-\prod_{u\leq t}\left(1-\Delta \hat{A}\left(u\right)\right)\\ & = & \sum_{u\leq t}\left(\prod_{v<u}\left(1-\Delta \hat{A}\left(v\right)\right)\Delta \hat{A}\left(u\right)\right)\\ & = & \sum_{u\leq t}\left(\prod_{v<u}\left(1-\Delta \hat{A}\left(v\right)\right)\Delta \hat{A_{1}}\left(u\right)\right)\\ & & \;+\;\sum_{u\leq t}\left(\prod_{v<u}\left(1-\Delta \hat{A}\left(v\right)\right)\Delta \hat{A_{2}}\left(u\right)\right). \end{array}$$

The first summand in the last equation of the previous display estimates P(T≤t,ε=1), the second summand estimates P(T≤t,ε=2). Both estimators depend on the estimation of both cumulative hazards via the “all‐events” Nelson–Aalen estimator $\hat{A}\left(t\right)$.

The example of competing events illustrates that independent censoring is about identifiability of hazards, but not necessarily about statements about the hypothetical situation of had there been no censoring. Censoring observed other deaths allows for identifiability of the hazard of cardiovascular death. But there is no reason whatsoever to consider this an analysis of a world where one may only die from cardiovascular reasons. Survival probabilities will, of course, depend on both event‐specific hazards. Andersen and Ravn (2024) offer further discussion on these aspects considering censoring by time ϑ as “formal” or “technical” censoring, which allows for analyzing event‐specific hazards. They contrast this with “avoidable” and “non‐avoidable” events, which is in line with our warning that not every censoring lends itself to “what, if?” considerations.

Summing up, the analysis of time‐to‐event data must account for censoring. Independent censoring as considered here is a more general concept than random censoring (possibly given covariates) that ensures identifiability of hazards. Identifiability of hazards does not necessarily imply an interpretation in a “world without censoring,” and probabilities may depend on multiple hazards.

However, readers are warned that there is an almost babylonian confusion on censoring concepts in the literature, where terms like “independent censoring,” “random censoring” and additionally “non‐informative censoring” are either used interchangeably—or are used to describe different censoring concepts, but not necessarily such that one term always describes the same censoring concept, see the discussion in Martinussen and Scheike (2006). We here follow Andersen et al. (1993) and Aalen et al. (2008), the latter giving a very accessible treatment, see also Andersen (2005).

## 3 Hazard Functions and Collider Bias

Hazards are conditional quantities, for example, Equation (3) was $\alpha \left(t\right)\;dt=P\left(T\in [t,t+\;dt)\;|\;T\geq t\right)$, and conditioning on previous survival T≥t was crucial to demonstrate that hazards remain identifiable in the presence of censoring. It is precisely conditioning on previous survival that gives rise to concerns about collider bias, which in turn has led to discussions about the utility or, at least, the interpretation of contrasting hazards to quantify effects even in the clear design framework of randomized interventions.

To illustrate, we consider a randomized intervention X∈{A, B} and we are interested in the intention‐to‐treat (ITT) effect of the intervention, decided upon at time 0, on time‐to‐death T∈(0,∞). Observation of T may be subject to random censoring by C, T⊥⊥C, but this is not central to the argument of concern. We here think of ITT not merely as a method of analysis, as may be the case in the current literature on estimands (e.g., Fletcher et al. 2022) along with critiques of “modified” ITT‐analyses. Rather, we think of ITT as a trial design principle. The reason is that we shall be interested in a “causal” intervention effect, which is formalized using randomization, and we consider the ITT effect, because it is the intention to treat that can be randomized. Because of randomization, the conditional probabilities P(·|X=A) and P(·|X=B) equal the interventional distributions indexed by the treatment intention, $P_{A}$ and $P_{B}$, respectively,

$$P\left(\cdot \;|\;X=A\right)=P_{A}\;\;\text{and}\;\;P\left(\cdot \;|\;X=B\right)=P_{B}.$$

The causal interpretation is that, for example, $P_{A}$ is the probability measure that holds if it is the intention to treat every unit or patient with treatment A. A common alternative formalization is via potential or counterfactual outcomes, where $P_{A}$ and $P_{B}$ are the marginal distributions of potential survival times under treatment A and B, respectively. We return to this alternative formalization in the discussion section.

A causal ITT effect on survival is now present iff, for the survival functions,

(9) $$\left(t\mapsto P(T>t\;|\;X=A)\right)\;\neq \;\left(t\mapsto P(T>t\;|\;X=B)\right),$$

where t is typically restricted to some [0,τ], τ<∞, for example, [0,τ] contained in the joint support of T and C. Note that (9) thus compares survival functions (on [0,τ]). In Section 2, we have argued that survival functions become identifiable via identifiability of their hazard functions, and of course, suppressing dependency on the intended treatment in the notation,

$$S\left(t\right)=P\left(T>t\right)=\exp\left(-\int_{0}^{t}\alpha \left(u\right)\;du\right)=\pi_{u\in (0,t]}\left(1-\;dA(u)\right),$$

illustrating the well‐known one‐to‐one relationship between survival function and hazard function, where π denotes the product integral. The Kaplan–Meier estimator introduced in Section 2 is then simply obtained from the product integral representation by estimating dA(u) by $\Delta \hat{A}\left(u\right)$ at the observed event times.

The consequence is that, if there is a difference in hazard functions, the presence of a causal ITT effect as in (9) follows. This is, for example, clearly stated by Martinussen (2022), but Martinussen also questions the interpretation of such a contrast between hazard functions as being a causal effect on the hazard. A related critique is in Hernán et al. (2004), who conclude that such a contrast is not a “direct effect of exposure on mortality.” The issue is collider bias (e.g., Pearl et al. 2016). The discussion often evolves around quantifying effects in terms of hazard ratios, but, as again Martinussen (2022) stresses, the core of the collider bias argument “is the hazard function itself, more than its particular structure.”

**FIGURE 1:**
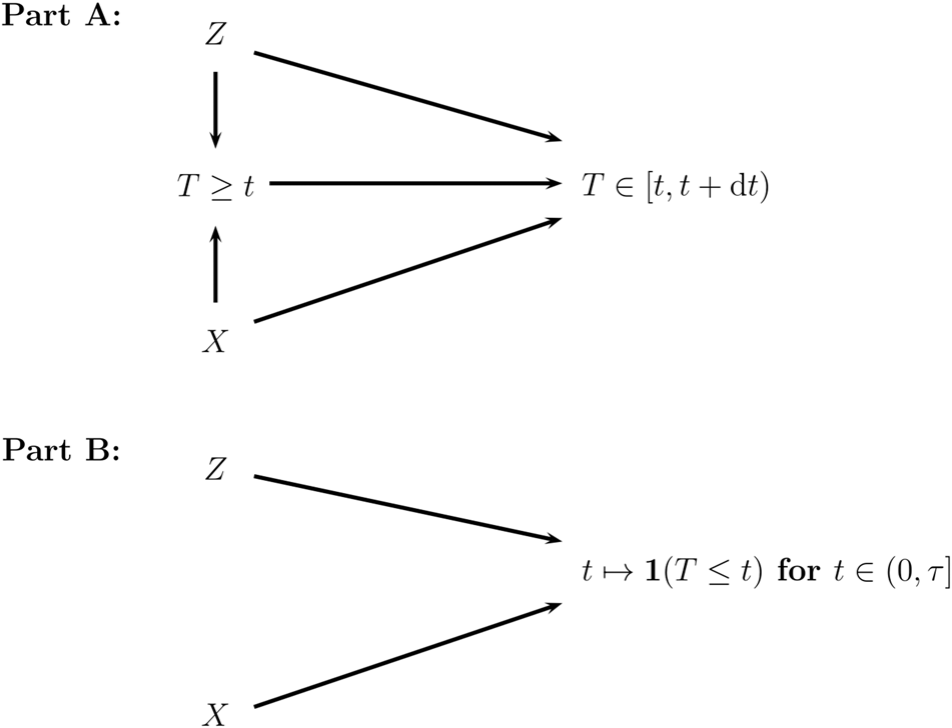
Part A: A directed acyclic graph with randomized treatment intention X, “confounder” Z and survival both prior time t and in the subsequent time step [t,t+dt). There are arrows originating from X and Z that collide in the intermediate node T≥t. Part B: A directed acyclic graph with X and Z as before and counting process outcome (0,τ]∋t↦1(T≤t). No arrows collide in an intermediate node.

Consider Figure 1A, variations of which can be found in Hernán et al. (2004) or Aalen et al. (2015). The figure is a directed acyclic graph whose arrows are to be interpreted as “has a causal effect on.” Survival both prior time t and in the subsequent time step [t,t+dt) is affected by both treatment intention X and some further baseline covariate and potential “confounder” Z, for example, disease stage, but there is no directed path from X to Z or vice versa nor do X and Z have a common ancestor. The interpretation is X⊥⊥Z and the reason is randomization.

Now, recall $\alpha \left(t\right)\;dt=P\left(T\in [t,t+\;dt)\;|\;T\geq t\right)$, where we condition on T≥t. Given this common child node in Figure 1A, X and Z will, in general, become dependent, X¬⊥⊥Z|T≥t. This is collider “bias,” the name stemming from the graph, where arrows originating from X and Z collide in T≥t. Given a common effect, survival prior t, two unconditionally independent causes become, in general, dependent. For instance, if X=A is protective, patients with values of Z leading to a poorer outcome will be more likely to survive up to t, if (intended to be) treated with A rather than with B (e.g., Schumacher et al. 1987). Note that this is the way it should be—and this might rather be collider “effect” than collider “bias”—and that this is not tied to time‐to‐event outcomes:

Assume, for the time being, that T is some continuous outcome in (0,∞), measured instantaneously without the need to wait for an event, and let us assume that larger outcomes are better. Considering, loosely speaking, the subset of units with a better outcome, that is, T≥t, there is no reason whatsoever to assume independence of treatment intention and confounder—for precisely the same reason as in the time‐to‐event case. If X=A is beneficial, patients with values of Z leading to a poorer outcome will be more likely to have outcomes of at least t if (intended to be) treated with A. In this case, one would not need to resort to hazards for data analysis, but it might be so that a treatment contrast would follow proportional hazards between treatment groups. The question still is how to causally interpret such a hazard contrast—recall the one‐to‐one relationship with probabilities—and to which extent it is affected by collider bias.

Figure 1A encodes causal effects of X and Z on the probabilities of T≥t and T∈[t,t+dt) and it is local in time. In fact, for a given t, realizations of X and Z must only precede t according to the graph, but what is really required investigating ITT effects is that X and Z are realized up to time 0. Therefore, consider Figure 1B, where the outcome node is the counting process (0,τ]∋t↦1(T≤t), which is in line with the causal contrast (9), and where both X and Z clearly precede (0,τ]. There is no collider bias in Figure 1B, and the causal ITT effect on the distribution of the outcome node (0,τ]∋t↦1(T≤t) may equivalently be expressed by (9) or by

(10) $$\left(t\mapsto \alpha (t\;|\;X=A)\right)\;\neq \;\left(t\mapsto \alpha (t\;|\;X=B)\right)\;\text{for}\;t\in \left(0,\tau \right].$$

The reason, of course, is the well‐known one‐to‐one relationship between survival function and hazard function mentioned earlier.

This does not state that there is no collider effect on the hazard, locally at time t. Suppressing dependency on the intended treatment in the notation, Equation (3) implies

(11) $$\alpha \left(t\right)\;dt=\frac{P\left(T\in [t,t+\;dt)\right)}{P(T\geq t)}.$$

There is, to the best of our knowledge, no discussion that there may be causal ITT effects on the unconditional probabilities in the numerator and in the denominator, respectively, on the right‐hand side of (11). The implication is that any ITT effect on α(t), interpreted locally in time, must be a mixture of such effects on the survival function P(T≥t) and, what Aalen et al. (2015) call “short‐term treatment effects,” on $P\left(T\in [t,t+\;dt)\right)$. It is because of the former that Hernán et al. (2004) deny that hazards can be used to describe “direct” effects, and we agree. (Note that the wording of Hernán et al. is more negative, stating that “[…] the hazard ratio […] is a biased estimate of the direct effect of exposure […].”) Another implication is that there can be an ITT effect on hazards because of the equality in (11), but the interpretation at the hazard level may be challenging and we advise to think of such effects as “kicking loose” a whole hazard function. The next Section 4 investigates this aspect in a bit more detail, while Section 5 considers a situation where the outcome node as in Figure 1B is multivariate.

## 4 Constant and Piecewise Constant Hazard Ratios

The previous section has stressed that a contrast of the hazard functions, that is, $\left(t\mapsto \alpha (t\;|\;X=A)\right)$ and $\left(t\mapsto \alpha (t\;|\;X=B)\right)$, has a causal ITT interpretation. Under a proportional hazards assumption,

(12) $$\frac{\alpha (t\;|\;X=B)}{\alpha (t\;|\;X=A)}=\exp\left(\beta \right)\;\text{for all}\;t\in \left(0,\tau \right],$$

and because this ratio compares functions, we have

(13) $$\exp\left(\beta \right)=\frac{\log\left(P_{B}\left(T>t\right)\right)}{\log\left(P_{A}\left(T>t\right)\right)}\;\text{for all}\;t\in \left(0,\tau \right],$$

as, for example, emphasized by Martinussen et al. (2020). We reiterate that there is no discussion about the comparison on the right‐hand side of Equation (13) being causal, as it contrasts unconditional interventional distributions. Martinussen (2022) questions the interpretation of exp(β) as quantifying a causal effect on the hazard at some time t, t>0, because of collider bias, but, as explained in the previous section, the issue of collider bias disappears if interpreted in a functional sense. To reiterate, the suggestion of the previous section is to compare the function t↦α(t|X=A) with t↦α(t|X=B) for t∈(0,τ]. This functional aspect also comes up in Vansteelandt et al. (2024), who therefore prefer ratios of cumulative hazards over hazard ratios and it is emphasized by Prentice and Aragaki (2022).

Still under model (12), De Neve and Gerds (2020) discuss an alternative interpretation of the hazard ratio on the probability scale using the probabilistic index. Assume that i and j denote two independent and randomly drawn units with exposure $X_{i}$ and $X_{j}$ and event times $T_{i}$ and $T_{j}$. Then

$$P\left(T_{i}<T_{j}\;|\;X_{i}=A,X_{j}=B\right)=\frac{1}{1+\exp(\beta )},$$

and the previous display has a causal interpretation, because it is equal to

$$-\int S_{B}\left(t\right)\;dS_{A}\left(t\right),$$

that is, can be expressed in terms of the interventional distributions, where we have again written S for the survival functions. De Neve and Gerds (2020) derive such a relation using counterfactuals, but this additional structure is not needed.

At this point, one could be tempted to let the question of interpreting exp(β) as a hazard ratio rest, having two alternative interpretations at the probability level available. However, a main worry in the influential paper by Hernán (2010) is arguably the interpretation of time‐varying hazard ratios. From the functional point of view of Section 3, a time‐varying hazard ratio still implies a causal contrast, but the point is more subtle and, to some extent, local in time. One modeling approach for time‐varying hazard ratios are period‐specific hazard ratios, constant within time‐periods. Traditionally viewed as displaying time‐varying effects, period‐specific reporting based on the event‐free population at the beginning of the period is certainly subject to a collider effect as explained earlier. However, the selection now is a consequence of period‐specific reporting, not tied to analyzing or reporting hazards.

**FIGURE 2:**
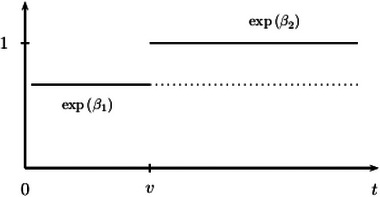
Piecewise constant hazard ratios as in (14) with $\exp\left(\beta_{2}\right)=1$. The dotted line corresponds to the hypothetical intervention $\tilde{B}$ discussed in the main text.

Martinussen et al. (2020) investigate this aspect in more detail, considering the model

(14) $$\frac{\alpha (t\;|\;X=B)}{\alpha (t\;|\;X=A)}=\left\{\begin{array}{cc} \exp\left(\beta_{1}\right) & \;\text{if}\;t\leq v,\\ \exp\left(\beta_{2}\right) & \;\text{if}\;t>v, \end{array}\right.$$

for some time v>0. If one of the hazard ratios above is equal to one and the other hazard ratio is less than one, this is typically interpreted as the treatment being ineffective on one time interval and beneficial on the other interval, see Figure 2. However, the situation is not symmetric. The interpretation is certainly correct, if $\exp(\beta_{1})=1$ and $\exp(\beta_{2})<1$, because the survival functions of the corresponding interventional distributions will then be equal on [0,v] and diverge afterwards. If, however, $\exp(\beta_{1})<1$ and $\exp(\beta_{2})=1$ as in Figure 2, Martinussen et al. (2020) “argue that there is no causal support” for such an interpretation. How does this comply with the functional point of view advocated earlier? Martinussen et al. (2020) build their argument on frailty modeling, which, on top of the subtle causal questions in time, brings in the issue of population versus individual hazards (Aalen et al. 2008, chapter 6). But the point can be made, and perhaps more accessibly so, at the population level:

Assume

$$\beta_{1}<0\;\;\text{and}\;\;\beta_{2}=0,$$

and note that the cumulative hazard in group B relates to that in group A as follows,

$$\begin{array}{cc} \int_{0}^{t}\alpha_{B}\left(u\right)\;du & =\exp\left(\beta_{1}\right)\int_{0}^{v\wedge t}\alpha_{A}\left(u\right)\;du\\ & \;+\exp\left(\beta_{2}\right)\left(\int_{0}^{t}\alpha_{A}\left(u\right)\;du-\int_{0}^{v\wedge t}\alpha_{A}\left(u\right)\;du\right). \end{array}$$

For t>v and using $\beta_{2}=0$, this leads to survival functions

(15) $$S_{B}\left(t\right)=\frac{{\left(S_{A}\left(v\right)\right)}^{\exp(\beta_{1})}}{S_{A}\left(v\right)}\cdot S_{A}\left(t\right)>S_{A}\left(t\right),$$

hence there still is a survival benefit on (v,∞) for the population intended to be treated with B at time origin. On the other hand, the left‐hand side of Inequality (15) can be re‐expressed as

(16) $$S_{B}\left(t\right)={\left(S_{A}\left(v\right)\right)}^{\exp(\beta_{1})}P_{A}\left(T>t\;|\;T>v\right),$$

where the last probability term again conditions on a collider, survival beyond v. We reiterate that period‐specific reporting produces its own selection, which is not specific to, say, period‐specific hazard ratios. The interpretation of (16) is that the intention to treat with B leads to a conditional survival probability given T>v equal to that in group A, but this does not mean that treatment is ineffective after time v. However, as is evident from both (15) and (16), the survival benefit after v is solely due to the hazard ratio being less than one on [0,v].

So, the situation $\beta_{1}<0$ and $\beta_{2}=0$ is not to be interpreted as the treatment initially being beneficial but ineffective later on the probability scale. But the situation of piecewise constant hazard ratios does differ from the setting where hazards are proportional throughout, and in this sense the treatment effect is time‐varying. To illustrate, contrast the situation at hand, that is, $\beta_{1}<0$ and $\beta_{2}=0$, with a hypothetical intervention, say $\tilde{B}$, with log‐hazard ratio equal to $\beta_{1}<0$ on (0,∞) when comparing $\tilde{B}$ with A. Of course, both B and $\tilde{B}$ lead to identical interventional survival functions on [0,v], which, however, diverge after v, with $\tilde{B}$ leading to higher survival probabilities due to or, one is tempted to write, because of $\beta_{1}<\beta_{2}$.

In summary, we suggest the following ITT interpretation for model (14) with $\beta_{1}<0=\beta_{2}$: The intention to treat with B leads to an interventional survival function that is superior to that of A, also after time v. The benefit after time v is a consequence of improved survival on [0,v]. However, the beneficial ITT effect on survival after v is less pronounced than in the case of a hypothetical treatment intention with log‐hazard ratio equal to $\beta_{1}$ for all times t. All this is succinctly expressed by model (14). Interpretation of the probabilistic index under model (14) appears to be less straightforward.

## 5 How Hazards Can Be Used to Understand, Why Probabilities Evolve the Way They Do

So far, we have argued that, first, hazards are the identifiable quantities in the presence of rather general censoring schemes and that, second, contrasts of hazard functions can be given a causal interpretation. We now consider two data examples on benefit assessment of drugs reported by Bender et al. (2016) that do not only illustrate that the first point is less well understood than should be the case. Benefit assessments are typically faced with more complex event histories and we will argue that they are best understood and, consequently, a causal ITT effect perhaps best formulated on the scale of hazard functions.

Bender et al. (2016) discuss two examples reported to the German Institute for Quality and Efficiency in Health Care (*Institut für Qualität und Wirtschaftlichkeit im Gesundheitswesen*, IQWiG for short) whose aim is to evaluate benefits and harms of medical interventions. The two examples consider randomized interventions of vandetanib for thyroid cancer and of abiraterone for prostate cancer, respectively. In both examples, time under the experimental treatment was prolonged as a consequence of a beneficial effect on progression‐free survival (PFS). Bender et al. report from dossiers turned in by the pharmaceutical companies (in German, URLs provided by Bender et al.). In a nutshell, companies reported statistically significantly increased proportions of observed adverse events, interpreted by companies to the disadvantage of the experimental drugs. The obvious shortcoming of such analyses—criticized by both Bender et al. and IQWiG—is that they both ignore possible censoring (see our discussion following Equation (1)) and different follow‐up times. A simple explanation for seeing more adverse events in the experimental treatment group could simply be prolonged PFS. The analyses using simple adverse event proportions were supplemented by estimating hazard ratios (either using Cox's model or a simple constant hazards model), which did not display a statistically significant increasing effect by the experimental treatment, now with the interpretation that harm had not been demonstrated.

For these two trials, we now track these (mis‐)interpretations in the English research literature (in contrast to the German dossiers mentioned above) and, more importantly, argue that an analysis of hazard functions disentangles matters. To begin, Wells Jr et al. (2012) report a pronounced protective effect on PFS in thyroid cancer patients treated by vandetanib with a hazard ratio of 0.46 (95% confidence interval [0.31,0.69]) in comparison to the control group. The authors also report increased proportions of observed adverse events, leading Solomon and Rischin (2012) to comment in an accompanying editorial that “*it is difficult to determine the extent to which the beneficial effects of vandetanib on tumor‐related symptoms and delays in tumor progression were counterbalanced by drug‐related adverse effects.”*’ A hazard‐based analysis of adverse events was not presented. We also note that the trial was event‐driven, see Wells Jr et al. (2012).

Ryan et al. (2013) report a pronounced protective effect on PFS in prostate cancer patients treated by abiraterone with a hazard ratio of 0.53 ([0.45,0.62]) in comparison to control. This trial was event‐driven, too. Adverse events were reported using simple proportions of observed events, leading the authors to summarize that certain adverse events were more common in the experimental arm. A hazard‐based analysis of adverse events was not presented. Rathkopf et al. (2014) present an updated analysis confirming the earlier results. Interestingly, the authors also report a hazard‐based analysis, finding graphically that “ *cumulative incidence of selected AEs (all grades) from the time of first event […] was similar across treatment groups*.” Unfortunately, the authors do not report their method to calculate incidences, but both the discussion of Bender et al. (2016), the reported large magnitude of, for example, cardiac disorders and no mentioning of competing events suggest that the reported curves are one minus a Kaplan–Meier–type transformation of the adverse‐event–specific Nelson–Aalen estimator.

A possible explanation of the picture at hand is as follows. Consider a time T to progression, death or adverse event, whatever happens first. Note that T<∞. Let us write $\alpha_{1}\left(t\right)$ for the event‐specific hazard for progression or death before an adverse event and $\alpha_{2}\left(t\right)$ for the event‐specific hazard for an adverse event before both progression and death. We argue that it would be natural to formalize that a treatment is beneficial w.r.t. progression/death while at the same time not being harmful w.r.t. adverse events as follows: Such a treatment decreases the hazard function $t\mapsto \alpha_{1}\left(t\right)$ and leaves the “competing” hazard function $t\mapsto \alpha_{2}\left(t\right)$ unchanged. The cumulative adverse event probability in this setting is

$$\begin{array}{cc} & \int_{0}^{t}P\left(T\geq u\right)\alpha_{2}\left(u\right)\;du\\ & \;=\int_{0}^{t}\exp\left(-\int_{0}^{u}\alpha_{1}\left(v\right)+\alpha_{2}\left(v\right)\;dv\right)\alpha_{2}\left(u\right)\;du. \end{array}$$

Hence, a treatment as just described increases the cumulative adverse event probability, because it increases P(T≥u) (via decreasing $t\mapsto \alpha_{1}\left(t\right)$), while leaving $t\mapsto \alpha_{2}\left(t\right)$ unchanged. (Note that the proper nonparametric estimator of the probability above is the Aalen–Johansen estimator explained in Section 2.)

The model just described is not perfect: Adverse events are typically recorded and analyzed until PFS plus an additional restricted follow‐up following progression (but, of course, not death) that leads to switch of treatment. PFS events are, of course, also recorded and analyzed after some prior adverse event. A more complete picture would therefore require an analysis of hazard functions for subsequent events, requiring methods for complex event histories (e.g., Aalen et al. 2008; Beyersmann et al. 2012). We also note that adverse events can also be recurrent and joint inference for a recurrent event process and mortality has, for example, recently been addressed by Furberg et al. (2022) and Furberg et al. (2023). But this is not the point here. Rather, the point is that hazard functions explain why and how event probabilities evolve over the course of time. If the aim of a randomized intervention is to detect ITT effects on event probabilities over the course of time, these can be detected and understood using hazard functions. Finally, we also note that the effect on hazard functions suggested by the two trials discussed above lends itself to a simpler interpretation than, say, a decreasing effect on $\alpha_{1}$ and an increasing effect on $\alpha_{2}$—or the other way around. For instance in the former case, an interpretation is that efficacy of the drug comes at the price of a worsened safety profile, and a summary in terms of all event probabilities is strongly suggested. We refer readers to Rufibach et al. (2024) as part of a special collection on the topic in *Trials* for further reading.

## 6 Discussion

We have argued that hazards have been and will continue to be key quantities for analyzing, interpreting, and understanding time‐to‐event data. They are needed in the statistical analysis, because hazards remain identifiable under rather general censoring schemes. We have also argued that the aspect of identifiability is less well appreciated than should be the case and that a general censoring concept beyond random censorship given covariates is needed. Of note, such general censoring does not necessarily lend itself to a causal interpretation of “what, if censoring can be avoided?” We also note that neither the usefulness of hazards nor subtleties when using them stop here. Hazards remain identifiable under independent delayed entry or left‐truncation and even under more general filtering concepts, see Erdmann et al. (2023) for a recent application to causal reasoning. Subtleties arise, because, for example, any time‐to‐event analysis requires a careful consideration of “time 0” and time‐dependent exposures may give rise to so‐called immortal time bias, which may again be avoided by a multivariate hazards analysis. The overview paper by Andersen et al. (2021) from the STRATOS initiative gives a very useful overview of such further aspects.

Our key contribution is that we have argued that hazard contrasts can be given a causal interpretation if interpreted in a functional sense. The debate about interpreting hazard contrasts causally often appears to be about hazard ratios, but the argument is about the conditional nature of hazards: Assuming a randomized intervention, conditioning on later survival is conditioning on a postrandomization event, leading to collider bias, or, as we have argued, collider effect. We have demonstrated that the collider effect vanishes in a functional sense. The conceptual implication is that we may very well envisage an intervention that causally impacts a population's future (postintervention) hazard functions, where the hazard measure may be multivariate. We illustrated matters with examples from benefit–risk assessment: Here, we found that censored data are not necessarily analyzed in a way that accounts for censoring. But if they are, the hazard analysis can well be interpreted in order to understand how hazard functions following an intervention lead to observed courses of disease. More specifically, we have argued that an effective drug that is safe in comparison may naturally be formalized as decreasing the hazard of the primary disease outcome while leaving the hazard of an adverse event unchanged.

In this line, we have also argued that piecewise constant hazard ratios—or general time‐varying hazard ratios, for that matter—carry information about how an intervention works, contrasting matters with a hypothetical intervention with a constant hazard ratio. Time‐varying hazard ratios have traditionally been interpreted as time‐varying effects. Whether such an interpretation is justified, is a major worry in the paper by Hernán (2010). Martinussen et al. (2020) argue that there is “no causal support” for such an interpretation. Again, the argument is the conditional nature of hazards and the ensuing collider effect. We argue that the situation is elusive, the question being what the notion of a “time‐varying effect” could really mean. The intuition appears to be that a treatment may work best initially, but its impact later decreases. Such reasoning is “flawed” from the current causal perspective, because it implicitly compares the future patient with the patient now. Translated onto the population level, the comparison is between one population at different time points and, as repeatedly stated earlier, such a comparison is not randomized anymore.

To elaborate further, assume we would accept the hazard function scale as a first approach to investigate possible time‐varying effects. An immediate question is: Is the effect truly time‐varying, or is the model simply misspecified? Assuming an additive model, say t↦t+β, with a time‐constant offset β>0 for the comparator group, the hazard ratio is time‐varying. Earlier, we have remarked that there is no debate about contrasts of unconditional survival functions being causal, but this is not a solution for the present question: A beneficial treatment is one that leads to higher survival probabilities, but all survival functions have value one at time zero and will eventually reach zero, denying immortality. Hence, contrasts of such survival functions will be time‐varying. The argument in our Section 4 suggests a third point of view: We detect a causal treatment effect of, say, treatment B by comparing it with some other treatment A. The comparison is essential. Settling on a contrast, say a hazard ratio, possibly time‐varying, we considered a hypothetical third treatment $\tilde{B}$, which initially had identical contrast compared with A, but a different contrast for later times. Here, it is meaningful to claim that the effect of B compared to $\tilde{B}$ varies over time, taking A as reference.

We have argued that the current debate is complicated by the fact that it often appears to be about hazard ratios, when the core of the argument is the hazard function itself, whatever the contrast. It is our impression that the elusiveness of what a time‐varying effect could be complicates matters further. To illustrate, Martinussen et al. (2018), in a preprint of their later paper, suggest that to “infer when treatment becomes ineffective, a multi‐stage design is ideally needed where patients on the treatment arm may randomly be switched to the control arm at designated points in time.” In contrast, Stensrud and Hernán (2020) argue that checking the proportional hazards assumption is unnecessary, because “[h]azards are not proportional when the treatment effect changes over time.” The former proposal does investigate a time‐varying effect via a time‐varying treatment plan, but not a time‐varying effect of the initial intention to treat. The latter statement is somewhat circular: It can only apply, if one both accepts hazards and their ratios as the scale to quantify treatment effects. It is arguably against this background that Martinussen (2022) calls for future research to find “a precise formulation of what is meant by” time‐varying effects. To this end, it is important to note that period‐specific reporting, be it of hazard ratios or of probabilities, will be subject to a collider effect if based on the event‐free population at the beginning of the period, and that this issue is not tied to the conditional nature of hazards.

Above, we have speculated that, intuitively, the notion of a time‐varying effect has one individual in mind, contrasting the patient now with the future patient. There are two other and more methodological aspects with connection to such a unit level that often feature in the current debate, but that we have avoided: To begin, frailties may be used to explicitly model the selection that occurs as a consequence of conditioning on a collider, see, in particular, Aalen et al. (2015) and Martinussen et al. (2020). In Section 3, we have chosen to not use frailty modeling, but to rely on general graphical criteria: If the joint distribution of the random quantities in a directed acyclic graph factorizes according to that graph, two random quantities with arrows colliding in an intermediate node will, in general, become conditionally dependent. As stated earlier, we have chosen to not resort to frailty arguments instead, because they bring in the issue of population versus individual hazards on top of the subtle causal questions at hand, and we have tried to not complicate matters further. As a consequence, we have stressed the population level rather than the individual level. It is well known that population hazards, in general, do not equal mean individual hazards, and frailty modeling is a very useful way to make this explicit. This arguably adds to the subtleties in the debate, as our notion about causality may well be at the individual level, while statistics rather operates at the population level. See, for example, Stensrud et al. (2019), who stress the connection between causal notions and the individual level and Balan and Putter (2020) for a tutorial on frailty models.

Such individual level also features when causality is formalized using potential outcomes or counterfactuals, which we have also avoided here. Focusing on a randomized intervention, we have used that the conditional probabilities equal the interventional distributions

$$P\left(\cdot \;|\;X=A\right)=P_{A}\;\;\text{and}\;\;P\left(\cdot \;|\;X=B\right)=P_{B}$$

and have contrasted

$$\left(t\mapsto P(T>t\;|\;X=A)\right)\;\;\text{with}\;\;\left(t\mapsto P(T>t\;|\;X=B)\right).$$

Counterfactual reasoning assumes the existence of a latent pair, say, $\left(T^{\left(A\right)},T^{\left(B\right)}\right)$ with

$$\left(T^{\left(A\right)},T^{\left(B\right)}\right)⊥\;\;\;⊥X,$$

because X is randomized, and

$$1\left(X=A\right)\cdot T^{\left(A\right)}+1\left(X=B\right)\cdot T^{\left(B\right)}=T.$$

Consequently, for example, for treatment A,

$$P\left(T>t\;|\;X=A\right)=P\left(T^{\left(A\right)}>t\;|\;X=A\right)=P\left(T^{\left(A\right)}>t\right),$$

that is, the interventional distribution equals the marginal distribution of the respective counterfactual. So far, the counterfactual formalization could be viewed as a matter of taste, although Occam's razor would favor the simpler formalization without counterfactuals. But the counterfactual formalization also allows statements beyond such a population level, directly contrasting $T^{\left(A\right)}$ with $T^{\left(B\right)}$. Such an individual causal contrast is “cross‐worlds,” because it is impossible to observe one individual's survival outcome under both A and B. In fact, it is not obvious how to formulate such “cross‐worlds” statements without counterfactuals. Dawid (2015) gives an insightful discussion that remains sceptical when it comes to counterfactuals.

Martinussen et al. (2020) use the counterfactual approach and consider contrasting hazards in the so‐called principal stratum of units fulfilling both $T^{\left(A\right)}>t$ and $T^{\left(B\right)}>t$. The definition of such a principal stratum fundamentally relies on assuming counterfactuals, but assuming both counterfactuals and the principal stratum to exist, and keeping time t fixed, the question of collider bias or collider effect disappears: Given this stratum, neither treatment X nor additional covariates Z impact survival on [0,t], because we have conditioned on the fact that the individual survives t whatever the treatment.

Earlier, a recurrent worry was conditioning on postrandomization survival. The “trick” of principal strata is to turn such a postrandomization problem into a prerandomization question by postulating the existence of a subgroup with both $T^{\left(A\right)}>t$ and $T^{\left(B\right)}>t$. The statistical task would be inference for such a subgroup that could at best be identified under strong assumptions. Perhaps more importantly, the interpretational question is the meaning one attaches to turning a postrandomization problem into a prerandomization question. See, for example, Dawid and Didelez (2012), Pearl (2011), and Stensrud and Dukes (2022) for further discussion. In particular, Stensrud and Dukes (2022) criticize that “principal strata are not observable when the [treatment] decision is made.” Here, our approach has been to consider the effect of the intention to treat on the whole population and argue that such a decision impacts future hazard functions, and that this may be expressed using hazard contrasts.

In conclusion, there is no debate that contrasts of unconditional (time‐to‐event) probabilities following a randomized intervention have a causal interpretation and there is hopefully no debate that such contrasts may be obtained from a hazard‐based analysis accounting for censoring. In line with the recent literature in the field, we also advocate that such transformations onto the probability scale are much more routinely conducted. This does not render interpretation of hazards and their contrasts useless. Interestingly, any paper questioning such interpretation as a consequence of the collider effect explains the selection over time and its consequences on hazards, similar to our Section 3, and, in a similar vein, for example, Aalen et al. (2015) see continued use of hazards when the aim is “understanding dynamic features of a complex event process.” It is in line with understanding the selection over time that hazard contrasts can be given a causal interpretation in the functional sense of Section 3, but perhaps equally important, they can be used to understand the contrasts of unconditional probabilities as demonstrated in Section 5. In our presentation, we have focused on the (“total”) effect of the randomized intention to treat, the reason being twofold: First, any other cause–effect relation is arguably even more complicated, and second, causal inference based on observational data formalizes a causal effect by trying to model or “mimic” a randomized intervention. We have also refrained from further interpreting the interventional effect on different hazard functions in Section 5. It would be tempting to interpret one effect as “direct” and one as “indirect,” and while this may be helpful in verbalizing how multivariate hazards lead to probabilities, there is no further causal interpretation at this stage. Such causal notions are particularly important when the aim is to investigate mediation and readers are referred to Didelez (2019) and Aalen et al. (2020). In this line, it is worthwhile to note that phenomena as discussed in Section 5 are by no means restricted to randomized trials, but do also occur in observational data, see Rojas‐Saunero et al. (2023) for a recent example from a causal perspective and Beyersmann et al. (2007) for a related discussion of associational aspects. Finally, the recent work of Røysland et al. (2024) investigates formal graphical criteria when the aim is to impose a new intensity by an intervention on a multivariate hazard measure.

## Conflicts of Interest

The authors have declared no conflicts of interest.

## Data Availability

Not applicable.
